# The impact of parenthood on Canadians’ objectively measured physical activity: an examination of cross-sectional population-based data

**DOI:** 10.1186/1471-2458-14-1127

**Published:** 2014-11-03

**Authors:** Anca Gaston, Sarah A Edwards, Amy Doelman, Jo Ann Tober

**Affiliations:** Brant County Health Unit, 194 Terrace Hill Street, Brantford, ON N3R 1G7 Canada; School of Kinesiology, Western University, 1151 Richmond Street, London, ON N6A 3K7 Canada

**Keywords:** Physical activity, Exercise, Sedentary lifestyle, Mothers, Fathers, Parents

## Abstract

**Background:**

Parenthood has been associated with declines in leisure-time exercise and moderate-to-vigorous physical activity (MVPA), but less is known about its impact on sedentary time and light-intensity activity. Although the health benefits of MVPA are well established, a growing body of research has been showing that even after controlling for MVPA levels, a detrimental dose–response association exists between sedentary time and adverse health outcomes and a beneficial dose–response association exists for light-intensity activity.

**Methods:**

This study examined the impact of parenthood, the number of children in the home, and the age of the youngest child on objectively measured physical activity (i.e., accelerometer derived daily minutes of sedentary, light, and MVPA) among a nationally representative cross-sectional sample of 2234 men and women who participated in the 2009–2011 Canadian Health Measures Survey.

**Results:**

After controlling for sociodemographic variables, ANCOVAs indicated that parents engaged in more light activity but less MVPA than non-parents and women whose youngest child was aged 12–15 years were more sedentary than women without children. Among both men and women, having a child <6 years of age in the home was associated with the greatest amount of light activity and lowest MVPA.

**Conclusions:**

Modest differences emerged between the physical activity level of parents and non-parents for both genders and across intensity levels. In general, parenthood was associated with less MVPA and more light-intensity activity, and more differences emerged among women compared to men. More research is needed before conclusions can be drawn regarding the health consequences of these differences.

## Background

Exercise participation has been shown to be negatively impacted by life transitions such as becoming a parent [1]. This is cause for concern, as parenthood is associated with weight gain and higher body mass index for both men and women [2]. Evidence also exists that parental inactivity may lead to inactivity among children, thus negatively impacting the health and well-being of future generations [2, 3].

In a meta-analytic review of 17 studies comparing the physical activity patterns of parents and non-parents, Bellows-Riecken and Rhodes [4] found that all but three studies reported a negative relationship between parenthood and exercise or physical activity. This difference was associated with an overall medium-sized effect and mothers were less active than fathers. Several more studies have been published since this review comparing physical activity levels between parents and non-parents with similar results. Hull et al. [5] found that adults who had a child reported a greater decrease in physical activity over the course of a 2-year period compared to those who remained childless. Similarly, Berge et al. [2] found that mothers engaged in less self-reported total exercise and both mothers and fathers engaged in less moderate-to-vigorous physical activity (MVPA) compared to non-parents. Using both self-report and accelerometer data, Candelaria et al. [6] found that parents spent more time engaged in household activities and less time sitting compared to non-parents. Parenthood was not related, however, to minutes of MVPA and self-reported leisure, transport, job-related, and total activity. Adamo et al. [7] analyzed cross-sectional accelerometer data collected as part of the 2007–2009 Canadian Health Measures Survey (CHMS) and found that mothers with children under age 6 and fathers with children aged 6–11 engaged in fewer minutes of MVPA per day compared to women or men without children. In addition, parents with children under age 6 were less likely to meet physical activity guidelines.

Only one study used accelerometry to examine intensity levels other than MVPA. In a prospective study, Rhodes et al. [8] compared changes in time spent in MVPA, light-intensity activity, and sedentary pursuits across 12 months among couples not expecting a child, expecting their first child, or expecting their second child. Parents who were expecting their second child engaged in fewer minutes of MVPA but were less sedentary and engaged in more light-intensity activity at baseline compared to couples without children. Compared to women without children, first-time mothers showed a larger decrease in minutes of MVPA across the study period.

Theoretical models can be useful in helping us understand physical activity behavior. One theoretical approach which may be particularly applicable to parenthood is the social-ecological model [4, 9, 10]. According to this approach, intrapersonal, interpersonal, the institutional and community environment, and policy factors all impact individuals’ ability or likelihood to engage in physical activity. In their review on parenthood and physical activity, Bellows-Riecken and Rhodes noted that despite the fact that parenthood is influenced by and embedded in all levels of this model, no study to date has explicitly adopted this approach [4]. After coding the studies in their review, these authors found that the majority focused only on intrapersonal and interpersonal factors while environmental and policy factors were excluded [4].

Although this body of evidence contributes towards our understanding of exercise participation during parenthood, it is not without limitations. Only three studies used accelerometry [6–8], and only one study examined intensity levels other than MVPA [8]. Most MVPA is comprised of leisure-time exercise and fitness activities such as brisk walking, jogging, cycling, and swimming [11]. Although the health benefits of MVPA are well established [12], a growing body of research has been showing that even after controlling for MVPA levels, a detrimental dose–response association exists between time spent sitting and adverse health outcomes, including cardiovascular disease mortality and all-cause mortality [13–15] and a beneficial dose–response association exists for light-intensity activity [16, 17]. Light-intensity activity characterizes most household activities (e.g., cooking, cleaning, doing laundry, gardening) and accounts for the majority of physical activity [11].

These findings highlight the importance of adopting an integrated approach which examines the distribution of activities in all intensity ranges across the 24-hour day [18]. Since an increase in one type of behavior necessarily requires a decrease in another type, the distribution of sleep, sedentary, light, and MVPA does play a role in the relationship between activity and health and/or disease. For example, using data from the US National Health and Nutrition Examination Survey, Buman et al. [18] demonstrated that beneficial associations were observed in cardiovascular disease risk markers with the reallocation of 30 minutes/day of sedentary time to either sleep, light-intensity activity, or, MVPA, which produced the strongest effect. Similarly, cluster analyses have shown that the accumulation of multiple risk factors is linked with poorer health outcomes compared to fewer risk factors (e.g., low MVPA and high sedentary vs. high MVPA and high sedentary) [13, 19, 20]. Although public health guidelines have traditionally focused solely on the promotion of MVPA, this, too, is starting to change. For example, in 2011, the Canadian Society for Exercise Physiology (CSEP) and the Healthy Active Living and Obesity Research Group of the Children’s Hospital of Eastern Ontario Research Institute released the first evidence-based integrated Canadian Sedentary Behaviour and Physical Activity Guidelines for children and youth ages 5–17 [21]. These guidelines stress the importance of taking a 24-hour approach and, in addition to accumulating at least 60 minutes of MVPA per day, recommend minimizing recreational screen time, motorized transportation, time spent sitting, and time spent indoors during the day [21]. According to CSEP, the development of similar guidelines aimed at adults is of immediate concern and will be developed as soon as resources are available [22].

### Purpose

While the existing research indicates that parenthood negatively impacts leisure-time exercise and MVPA, further research is needed to understand the relationship between parenthood and overall activity patterns (i.e., sedentary behavior, light-intensity activity and MVPA) and inform the need for population-level interventions across the activity spectrum [23]. Thus, the purpose of the present study was to examine the impact of a number of interpersonal factors related to parenthood (i.e., parental status, the number of children in the home, and the age of the youngest child) on objectively measured physical activity across intensity levels (i.e., daily minutes of sedentary, light, and MVPA) among a nationally representative sample of men and women between the ages of 18 and 64 participating in the 2009–2011 CHMS.

We hypothesized that parents would engage in less MVPA, more light-intensity activity, and less sedentary activity. These hypotheses were based on evidence from cross-sectional studies indicating that parents tend to engage in less leisure-time physical activity compared to non-parents [4] and from prospective studies indicating that physical activity decreases from pre-parenthood to parenthood [5, 8, 24].

## Methods

### Data source

The CHMS is a cross-sectional, nationally-representative survey covering household populations aged 3 to 79 [25]. The survey is conducted by Statistics Canada with ethical approval granted by Health Canada and the Public Health Agency of Canada. The survey excludes residents of Indian Reserves or Crown lands, institutions, certain remote regions, and full-time members of the Canadian Forces. It represents approximately 96% of Canadians and each survey respondent is given a weight corresponding to the number of persons represented by the respondent. The survey is voluntary and data were collected at 18 sites across Canada between August 27, 2009 and November 30, 2011 (Cycle 2). In addition to a personal interview, participants also underwent a series of direct measures at a mobile examination unit and received an Actical accelerometer (Phillips – Respironics, Oregon, USA) to wear. Participants were instructed to wear the accelerometer during all waking hours for 7 days starting the day after their mobile examination centre appointment. More information about the CHMS is available through Statistics Canada’s Web site (http://www.statcan.ca).

### Population

Parents were defined as adults living with a dependent child aged 15 years or younger. The CHMS (Cycle 2) dataset included 6395 respondents, of which 4948 (72.7%) provided valid accelerometer data. Pregnant women (n = 23), respondents which did not provide complete demographic data (n = 59), parents living with a dependent child aged 16 or older (n = 159), and respondents who were not between the ages of 18 and 64 (n = 2473) were excluded. In total, 2234 respondents (1205 women and 1029 men) were eligible for the current study. Five hundred eighty-six women (48.6%), and 440 (42.8%) men had at least one dependent child aged 15 or younger in the home.

### Accelerometer data

The Actical is a small, lightweight waterproof, omni-directional accelerometer which is worn over the right hip using an elasticized belt. All data are blind to participants while the device is being worn. A 1-minute epoch was used and monitors were calibrated prior to the start of data collection and re-tested upon return. In line with previous population-based analyses using accelerometry [26], a day had to include 10 or more hours of wear time in order to be considered valid, and only respondents with 4 or more valid days were included in analyses. Accelerometer measurement and data treatment procedures have been described in full elsewhere [26].

### Parenthood variables

Parental status was determined based on a single derived variable describing the living arrangement of the respondent. Respondents categorized as ‘Parent living with spouse/partner and children’ or ‘Single parent living with children’ were classified as ‘parents,’ and respondents in all other living arrangements were categorized as ‘non-parents.’ The variable ‘Number of persons in household less than 16 years of age’ was used to ensure that parents had at least one child in the household under the age of 16 and the number of children in the home (i.e., none, 1, 2, or 3 or more). In addition, the variables ‘Number of persons in household less than 12 years of age’ and ‘Number of persons in household less than 6 years of age’ were used to determine the age category of the youngest child in the home (i.e., <6 years, 6–11 years, or 12–15 years).

### Dependent variables

The variables of interest for this study were: average daily minutes of sedentary, light, and MVPA. Validated cut-points were applied to the raw accelerometer data in order to convert the raw counts per minute (cpm) into corresponding intensity levels: sedentary (1 to less than 2 metabolic equivalents (METS); <100 cpm), light (2 to less than 3 METS; 100 – 1534 cpm), and MVPA (3 or more METS; 1535 or more cpm) [26].

### Covariates

Evidence exists that physical activity may be associated with numerous intrapersonal (e.g., demographic) characteristics such as age, body mass index (BMI), ethnicity, income, education, marital status, and health status [27, 28]. For this reason, these demographic characteristics were used as covariates. Age and BMI were treated as continuous variables. Marital status was categorized as married/common-law versus Single/separated/widowed/divorced. Highest education level achieved was grouped into less than secondary, secondary school diploma, some post-secondary, or post-secondary graduate. Total household income, adjusted for the number of people in the household, was categorized into lowest income grouping, lower middle income grouping, upper middle income grouping, and highest income grouping. Ethnicity was collapsed into white versus other, and self-rated health was categorized as poor/fair, good, very good, and excellent. Except for self-rated health, which was collapsed from five to four categories because of limited sample sizes in the poor and fair categories, variable categories reflect CHMS response options.

### Statistical analyses

All analyses were conducted separately by gender and using weighted data to correct for non-response bias and to represent the Canadian population. This weight corresponds to the number of individuals in the entire population which are represented by each respondent. First, descriptive statistics (means and 95% confidence intervals) were computed for parents and non-parents with respect to demographic and personal characteristics as well as daily minutes of sedentary, light, and MVPA. Next, one-way ANCOVAs were used to examine whether group differences existed for parenthood status (i.e., parents versus non-parents), age of youngest child (i.e., <6 years, 6–11 years, or 12–15 years), or number of children (i.e., none, 1, 2, or 3 or more) with respect to the variables of interest: sedentary, light, or MVPA per day. All analyses controlled for parental age, marital status, education, income, ethnicity, self-rated health, and BMI. Variance estimates (95% confidence intervals) and significance tests were calculated using bootstrap procedures. ANCOVAs were followed up with pairwise comparisons using a Bonferroni correction. All statistical analyses were conducted using IBM SPSS version 21.0 during September to October 2013.

## Results

### Participants

This study included 2234 men and women representative of the Canadian population between the ages of 18 and 64. Irrespective of gender, parents tended to be significantly younger, married or living with a common-law spouse, more likely to have attained post-secondary education, and less likely to report having poor or fair health compared to non-parents. The demographic characteristics of the sample are reported in Table 1.

**Table 1:** **Descriptive characteristics of the sample, by gender and parenthood status**

	Women (n =1205)				Men (n =1029)			
	Parent of at least one dependent child				Parent of at least one dependent child			
	No (n =619)		Yes (n =586)		No (n =589)		Yes (n =440)	
	n	% (95% CI)	n	% (95% CI)	n	% (95% CI)	n	% (95% CI)
Age (years)^a^								
18-34	191	30.9 (27.1, 34.6)	205	35.0 (31.2, 38.7)	194	32.9 (29,0,36.7)	115	26.1 (22.3, 29.8)
35-49	133	21.5^*^ (17.9, 24.9)	358	61.1 (57.2, 65.0)	137	23.3^*^ (20.0, 26.5)	291	66.1 (61.8, 70.4)
50-64	295	47.7^*^ (43.8, 2.0)	23	3.9 (2.6, 5.6)	258	43.8^*^ (39.7, 47.7)	34	7.7 (5.2, 10.2)
Marital status								
Single/divorced/separated/widowed	318	51.4^*^ (47.3, 55.4)	94	16.0 (13.1, 19.1)	325	55.2^*^ (51.1, 58.9)	14	3.2 (1.6, 4.8)
Married/common-law	301	48.6^*^ (44.6, 52.7)	492	84.0 (80.9, 86.9)	264	44.8^*^ (41.1, 48.9)	426	96.8 (95.2, 98.4)
Education (highest achieved)								
< Secondary	63	10.2 (7.9, 12.8)	37	6.3 (4.6, 8.4)	66	11.2 (8.8, 14.1)	33	7.5 (5.2, 10.0)
Secondary school diploma	107	17.3 (14.5, 20.2)	66	11.3 (8.9, 13.8)	92	15.6 (12.7, 18.7)	60	13.6 (10.7, 17.0)
Some post-secondary	73	11.8^*^ (9.2, 14.4)	37	6.3 (4.4, 8.4)	86	14.6^*^ (11.7, 17.7)	19	4.3 (2.7, 6.4)
Post-secondary graduate	376	60.7^*^ (57.0, 64.8)	446	76.1 (72.7, 79.5)	345	58.6^*^ (54.3, 62.3)	328	74.5 (70.0, 78.4)
Income adequacy								
Lowest income	37	6.0 (4.2, 7.9)	40	6.8 (4.9, 8.9)	31	5.3 (3.6, 7.0)	10	2.3 (0.9, 3.9)
Lower middle	80	12.9 (10.5, 16.0)	79	13.5 (10.9, 16.4)	63	10.7 (8.3, 13.4)	46	10.5 (8.0, 13.4)
Upper middle	198	32.0 (28.3, 35.7)	182	31.1. (27.1, 35.0)	170	28.9 (25.0, 32.8)	125	28.4 (24.3, 32.7)
Highest income	304	49.1 (45.2, 53.3)	285	48.6 (44.9, 52.9)	325	55.2 (50.9, 59.3)	259	58.9 (54.3, 63.6)
Ethnicity								
White	511	82.6 (79.3, 85.5)	456	77.8 (74.4, 80.9)	485	82.3 (79.1, 85.6)	352	80.0 (76.1, 84.1)
Other	108	17.4 (14.5, 20.7)	130	22.2 (19.1, 25.6)	104	17.7 (14.4, 20.9)	88	20.0 (15.9, 23.9)
Self-rated health								
Poor/fair	63	10.2^*^ (8.1, 12.6)	25	4.3 (2.7, 6.1)	63	10.7^*^ (8.3, 13.2)	18	4.1 (2.3, 6.1)
Good	227	36.7 (33.1, 40.4)	204	34.8 (31.2, 38.4)	201	34.1 (30.2, 37.9)	150	34.1 (29.8, 38.6)
Very good	245	39.6 (35.4, 43.5)	245	41.8 (37.5, 45.7)	218	37.0 (32.9, 40.9)	201	45.7 (40.9, 50.4)
Excellent	84	13.6 (10.8, 16.3)	112	19.1 (16.0, 22.4)	107	18.2 (15.1, 21.4)	71	16.1 (12.7, 19.5)
BMI classification^a^								
Normal or underweight	286	46.2 (42.0, 50.1)	295	50.3 (46.2, 54.8)	208	35.2 (31.4, 39.4)	122	27.7 (23.4, 31.8)
Overweight	178	28.8 (25.0, 32.5)	167	28.5 (25.1, 32.1)	246	41.8 (37.5, 45.7)	204	46.4 (41.8, 51.1)
Obese	155	25.0 (21.6, 28.6)	124	21.2 (17.9, 24.6)	135	22.9 (19.9, 26.5)	114	25.9 (21.6, 30.2)

*Note.* BMI = body mass index (weight in kilograms divided by height in metres squared) ^a^Age and BMI are presented as categorical variables for descriptive purposes only. For covariate purposes, these variables were continuous.

^*^Significantly different from respondents of the same gender with children (*p* < .05).

### Physical activity and parenthood status

#### Women

Women with at least one dependent child in the home engaged in significantly fewer minutes of daily MVPA (*p* = .000) but significantly more minutes of light intensity activity (*p* = .001) compared to women without any dependent children in the home (see Table 2). When number of children was examined, women with one child engaged in more light-intensity activity (*p* = .005) than women without children, and women with two or three or more children engaged in fewer minutes of MVPA (*p*s = .000 and .005, respectively) compared to women without children. Finally, when the age of the youngest child was examined, women whose youngest child was aged 12–15 years engaged in significantly more sedentary behavior than women without children (*p* = .017), whereas women whose youngest child was aged six years or younger engaged in more light-intensity activity (*p* = .000), and less MVPA (*p* = .000) compared to women without children.

**Table 2:** **Average minutes of daily physical activity by intensity and parenthood status for women**

		Intensity		
		Sedentary	Light	MVPA
Variable	n	Mean (95% CI)	Mean (95% CI)	Mean (95% CI)
Parent of a dependent child under 16 years				
No	619	595.1 (582.1, 606.5)	208.7 (197.9, 220.2)	20.2 (17.9, 22.8)
Yes	586	586.4 (570.4, 599.9)	251.2^***^ (245.6, 257.0)	17.8^***^ (16.6, 18.9)
ANCOVA *p*-value		.263	.001	.000
Effect size (*η* ^2^)		.001	.009	.013
Number of children under 16 in home				
None	619	595.1 (582.1, 606.5)	208.7 (197.9, 220.2)	20.2 (17.9, 22.8)
1	211	582.4 (559.5, 603.1)	244.9^**^ (225.6, 263.5)	19.0 (15.1, 23.3)
2	294	587.8 (567.1, 605.8)	233.8 (219.2, 250.6)	16.2^***^ (13.0, 20.3)
3 or more	81	597.7 (556.6, 639.0)	251.4 (232.0, 280.3)	12.3^**^ (7.8, 18.1)
ANCOVA *p*-value		.213	.005	.000
Effect size (*η* ^2^)		.004	.011	.019
Age of youngest child in home				
0 (no children)	619	595.1 (582.1, 606.5)	208.7 (197.9, 220.2)	20.2 (17.9, 22.8)
<6	339	580.8 (562.5, 600.0)	255.2^***^ (239.8, 270.2)	14.6^***^ (12.0, 17.7)
6-11	215	577.9 (547.6, 606.3)	235.6 (214.3, 259.4)	21.1 (15.5, 27.2)
12-15	32	621.8^*^ (593.8, 642.8)	209.1 (189.8, 232.9)	16.7 (11.9, 21.9)
ANCOVA *p*-value		.017	.000	.000
Effect size (*η* ^2^)		.008	.016	.036

*Note.* Asterisks signify significant post-hoc differences (Bonferroni corrected) between respondents in the target category and those without children. ANCOVAs controlled for age, body mass index, marital status, education, ethnicity, income adequacy, and self-rated health. All estimates rounded to one decimal point based on Statistics Canada’s rounding guidelines in order to avoid implying greater precision than actually exists. CI = Confidence interval. MVPA = moderate-to-vigorous physical activity.

^*^ *p <* .05. ^**^ *p <* .01. ^***^ *p <* .001.

#### Men

Among men, the presence of a dependent child in the home was related only to light activity, so that men with a dependent child in the home engaged in more light intensity activity than their counterparts without children (*p* = .000; see Table 3). Light activity was similarly the only intensity related with the number of children in the home, so that fathers with 2 children in the home engaged in more light intensity activity than men without children (*p* = .000). With respect to the age of the youngest child in the home, men with a child younger than six years of age engaged in more light activity (*p* = .000) but less MVPA than men without children (*p* = .000).

**Table 3:** **Average minutes of daily physical activity by intensity and parenthood status for men**

		Intensity		
		Sedentary	Light	MVPA
Variable	n	Mean (95% CI)	Mean (95% CI)	Mean (95% CI)
Parent of a dependent child under 16 years				
No	589	579.4 (567.2, 593.6)	230.6 (218.6, 243.1)	27.5 (23.5, 31.8)
Yes	440	573.4 (565.0, 582.6)	265.7^***^ (258.0, 273.0)	24.1 (22.2, 26.5)
ANCOVA *p*-value		.212	.000	.599
Effect size (*η* ^2^)		.002	.016	.000
Number of children under 16 in home				
None	589	579.4 (567.2, 593.6)	230.6 (218.6, 243.1)	27.5 (23.5, 31.8)
1	125	582.3 (547.8, 610.7)	249.2 (223.0, 271.4)	23.7 (17.5, 32.4)
2	231	567.7 (552.2, 585.0)	267.1^***^ (252.3, 282.1)	21.9 (19.1, 25.2)
3 or more	84	568.7 (548.7, 592.7)	257.6 (237.0, 281.0)	20.4 (17.7, 24.6)
ANCOVA *p*-value		.314	.000	.800
Effect size (*η* ^2^)		.003	.018	.001
Age of youngest child in home				
0 (no children)	589	579.4 (567.2, 593.6)	230.6 (218.6, 243.1)	26.4 (22.9, 30.3)
<6	264	563.0 (544.1, 581.2)	270.4^***^ (255.6, 287.7)	20.2^*^ (16.4, 24.6)
6-11	150	580.1 (564.3, 597.2)	252.0 (235.9, 269.1)	28.3 (23.6, 33.1)
12-15	26	594.7 (553.9, 625.8)	233.11 (199.1, 269.2)	35.5 (22.7, 53.0)
ANCOVA *p*-value		.404	.000	.000
Effect size (*η* ^2^)		.003	.019	.019

*Note.* Asterisks signify significant post-hoc differences (Bonferroni corrected) between respondents in the target category and those without children. ANCOVAs controlled for age, body mass index, marital status, education, ethnicity, income adequacy, and self-rated health. All estimates rounded to one decimal point based on Statistics Canada’s rounding guidelines in order to avoid implying greater precision than actually exists. CI = Confidence interval. MVPA = moderate-to-vigorous physical activity.

^*^ *p <* .05. ^***^ *p <* .001.

## Discussion

This is the first study to examine the impact of parenthood on Canadians’ intensity-specific physical activity using accelerometers and a nationally representative sample. Differences emerged between the physical activity level of parents and non-parents for both genders and across intensity levels. As hypothesized, parents seem to engage in more light-intensity activity but less MVPA compared to non-parents, and parenthood appears to be more strongly related to physical activity among women compared to men. Cohen [29] recommended using the following values to interpret the strength of the eta-squared effect: .01 small, .06 moderate, and .14 large. Effect sizes using this criterion reveal small differences for light-intensity activity among both men and women and small to small-moderate sized differences for MVPA among men and women, respectively.

Light-intensity activity was positively related to parenthood, number of children, and age of youngest child for both women and men. These results are in line with Candelaria et al. [6] and Rhodes et al.’s [8] findings. Specifically, Candelaria et al. [6] found that parents engaged in more self-reported household activity than non-parents and Rhodes et al. [8] found that parents engaged in more objectively-measured light-intensity activity than non-parents, a difference equivalent to a moderate effect size. Although the effect sizes in the present study were small, the findings are encouraging nonetheless because convincing evidence exists that even after controlling for sedentary time and MVPA, engaging in light-intensity activity is associated with numerous positive outcomes including lower cardiovascular heart disease rates [16], improved blood glucose levels [17], lower waist circumference and reduced metabolic risk [30], and improved physical health and well-being [31]. Light-intensity activities include the majority of household tasks, such as cooking, washing dishes, laundry, and light gardening and are the major determinants of variability in total energy expenditure because of the number of daily hours they represent [11]. Having one child was associated with more light activity for women whereas having 2 children was associated with the most activity among men. This is consistent with findings that having 2 children in the home is the only family configuration associated with a reduction in men’s recreation time [32]. For both sexes, having at least one child under the age of six was associated with more light activity. This is consistent with evidence indicating that the younger the child the more parental time is spent on caregiving activities [33].

For women, being a mother, having 2 or more children, or a child under 6 were all associated with lower MVPA (small to small-moderate effect sizes) whereas among fathers, only having a child under 6 was associated with lower MVPA (small effect size). These findings are in line with previous research indicating that women’s participation in leisure-time exercise is more adversely affected by parenthood than men’s [4]. However, the present effect sizes were smaller than the moderate effect sizes reported by Rhodes et al. [8] using accelerometer data and Bellows-Riecken and Rhodes [4] in their review on parenthood and physical activity. It should also be noted that MVPA levels were low irrespective of gender and parenthood status. While this confirms previous findings [26], it is cause for concern as the health benefits associated with regular MVPA are well-established and include reduced all-cause morbidity and mortality [12].

In contrast to our hypotheses and previous research [6, 8], parenthood was not consistently related to sedentary time. Only one difference emerged, such that women whose youngest child was 12 to 15 years old spent more time being sedentary compared to women without children. However, this finding should be interpreted with caution based on the small number of women with children in this age range. Sedentary time refers to almost all sitting-based activities and includes work-related sitting as well as recreational-based sedentary activities such as watching television, reading, or socializing [11, 34]. Irrespective of parenthood, men and women spent over nine and a half hours of their day in sedentary pursuits, a finding which is of concern given the public health burden associated with excessive sitting [13–15].

In addition, it should be pointed out that when time spent in sedentary, light, and MVPA intensity activity is summed, it becomes clear that parents accumulated approximately 30 more minutes of wear time per day compared to non-parents. Since all participants wore the accelerometer for all waking hours, this difference indicates that parents spent more time awake (i.e., slept less) compared to non-parents. This finding is in line with previous research. For example, a study analyzing data collected from over 19,500 respondents representing 26.1 million Canadians as part of Statistics Canada's 2005 General Social Survey found that “kids deprived parents of sleep” [35]. Specifically, Canadians with two or more children slept, on average, 25 minutes less than Canadians without children [35]. From our results, it appears that Canadian parents spend the time they are not sleeping engaged in light-intensity activities, most likely related to their additional caregiving responsibilities. While the reallocation of sedentary time to sleep, light-intensity activity or MVPA appears to be beneficial [18], the health consequences of reallocating sleep time to light-intensity activity are unknown. Nevertheless, from a physical activity standpoint it is encouraging to find that this additional ‘waking time’ was not spent being sedentary (e.g., watching television).

From a social ecological perspective, there are a number of reasons why the present differences may have emerged. Personal factors such as time constraints and a lack of money due to the responsibilities and costs associated with raising children are both associated with lower exercise participation and may explain why parents tend to engage in less MVPA or leisure-time exercise [36]. Several organizational, environmental and policy factors have also been shown to negatively affect physical activity. These include a societal pressure to be a good parent, volunteer, and fundraise for their child’s school, work and social cultures that don’t value physical activity, work demands which do not make allowances for parenthood, and rigid work hours [36]. These factors also help explain why parenthood was associated with more light-intensity activity but less MVPA, and why greater differences were observed for women compared to men. Specifically, evidence exists that compared to fathering, mothering is associated with a greater overall time commitment, more physical work, more time alone with children, more overall responsibility, and, not surprisingly, less free time [37]. With respect to income, motherhood is associated with lower hourly pay, resulting in mothers having less money to spend on themselves compared to fathers [38].

With respect to research, these findings underscore the importance of examining overall patterns of physical activity rather than only MVPA. Historically, reliance on self-reported data has prevented these types of investigations as questionnaires lack the sensitivity to accurately quantify light-intensity activity and sedentary time [11]. As more researchers begin to incorporate accelerometers into their work, there is no doubt that more studies will begin to take a more comprehensive view of physical activity by examining intensities beyond MVPA.

In terms of practical significance, the present results highlight the importance of continuing to promote MVPA participation among both parents and individuals without children. Health habits are established during childhood and by making the time to exercise, parents can act as important role models for their children [2]. With respect to light activity, parents should be encouraged to continue being as active as possible. Our findings also highlighted the fact that all participants spent the largest proportion of their day being sedentary. Given the adverse health outcomes associated with high sedentary time [39], public health interventions aimed at reducing sedentary behavior are justified.

Strengths of the present study include objectively measured physical activity data from participants representative of the Canadian population. Analyses were conducted separately to examine the impact of parenthood, number of children, as well as the age of the youngest child, allowing us to more closely examine the relationship between physical activity and parenthood. However, several limitations must also be acknowledged. First, the cross-sectional design of the CHMS precludes us from drawing any cause and effect conclusions about the relationship between parenthood and physical activity. As parents and non-parents may be different in a number of ways, including, for instance, in their degree of interest in physical activity, our inability to control for additional ‘third factors’ represents a limitation. More studies employing prospective designs are needed to draw stronger conclusions regarding the impact of parenthood on physical activity levels. Second, the way in which the CHMS assessed living arrangements may have impacted our ability to correctly categorize all individuals as parents versus non-parents. For example, for participants who indicated that they were a ‘single parent living with children,’ there was no way to know what percentage of time the children actually lived with them. As a result, some individuals may have been categorized as ‘parents’ even though they were not the main caregivers of a child. However, only 16.0% of women and 3.2% of men in our sample were single parents (see Table 1), making it unlikely that this limitation could have significantly impacted the present results. Finally, this analysis only includes participants with complete accelerometer data (72.7% of the original CHMS sample). However, each respondent in this subsample was assigned a weight corresponding to how many individuals from the general population they represent in order to minimize response bias.

## Conclusions

In summary, Canadian parents consistently engage in more light-intensity physical activity but less MVPA than non-parents. Women’s MVPA levels appear to be more strongly associated with parenthood than men’s. For both parents, having a child under 6 was associated with the lowest levels of MVPA but the highest levels of light intensity activity. Although evidence exists suggesting that optimal health depends as much upon reducing sedentary time and increasing light-intensity activity as it does upon accumulating adequate MVPA, more research is needed before conclusions can be drawn regarding the health effects associated with these differences.

## Abbreviations

ANCOVA: Analysis of covariance
BMI: Body mass index
CHMS: Canadian health measures survey
Cpm: counts per minute
METS: Metabolic equivalents
MVPA: Moderate-to-vigorous physical activity.
